# Insights Into the Future: Assessing Medical Students' Artificial Intelligence Readiness ‐ A Cross‐Sectional Study at Kerman University of Medical Sciences (2022)

**DOI:** 10.1002/hsr2.70870

**Published:** 2025-05-26

**Authors:** Hossein Rezazadeh, Ali Madadi Mahani, Mahla Salajegheh

**Affiliations:** ^1^ Student Committee of Medical Education Development, Education Development Center Kerman University of Medical Sciences Kerman Iran; ^2^ Department of Medical Education, Medical Education Development Center Kerman University of Medical Sciences Kerman Iran

**Keywords:** artificial intelligence, medical education, readiness

## Abstract

**Background:**

Artificial intelligence (AI) has recently advanced in medicine globally, transforming healthcare delivery and medical education. While AI integration into medical curricula is gaining momentum worldwide, research on medical students' preparedness remains limited, particularly in developing countries. This paper aims to investigate the readiness of medical students at the Kerman University of Medical Sciences to employ AI in medicine in 2022.

**Methods:**

This cross‐sectional research was carried out by distributing the validated 20‐item Medical Artificial Intelligence Readiness Scale for Medical Students (MAIRS‐MS) among 360 medical students, with a response rate of 94% (*n* = 340). The MAIRS‐MS assessed four domains, including cognition (8 items), ability (7 items), vision (2 items), and ethics (3 items), using a 5‐point Likert scale. Data analysis was conducted by descriptive statistics and independent sample *t*‐tests in SPSS v24.0, considering *p* < 0.05 significant.

**Results:**

Participants demonstrated below‐average readiness scores across all domains: ability (*M* = 21.88 ± 6.74, 62.5% of the maximum possible score), cognition (*M* = 20.30 ± 7.04, 50.8%), ethics (*M* = 10.94 ± 3.04, 72.9%), and vision (*M* = 6.09 ± 1.94, 60.9%). The total mean readiness score was 59.21 ± 16.12 (59.2% of the maximum). The highest and lowest‐rated items were “value of AI in education” (3.96 ± 1.18) and “explaining AI system training” (2.10 ± 1.01), respectively. No significant differences were found across demographic factors (*p* > 0.05).

**Conclusion:**

Iranian medical students currently show limited readiness for AI integration in healthcare practice. Therefore, the study recommends: (1) implementing structured introductory AI courses in medical curricula, focusing particularly on technical fundamentals and practical applications, and (2) developing hands‐on training programs that combine AI concepts with clinical scenarios. These findings provide valuable insights for curriculum development and educational policy in medical education.

## 1 Introduction

Artificial intelligence (AI) has recently found extensive applications in medical practice [1]. Today, artificial intelligence‐based health/medical care programs can assist physicians in various ways, such as supporting vigorous machine learning algorithms in medical imaging, comprising X‐ray, CT scan, and MRI diagnosis, acoustic data‐based pattern recognition and disease prediction, and prognosis on disease types and developmental trends for patients [2]. For instance, the disease diagnosis is facilitated when AI analyzes electrocardiograms [3], providing information on the patients' age, gender, anemia, risk of diabetes or arrhythmias, heart function, valve complications, and kidney or thyroid conditions [4]. AI can also contribute to identifying psychotic and neurological illnesses, including Parkinson's disease using speech patterns [5], detecting polyps and neoplasms in the digestive system [6], and even automating surgical tasks and enhancing intraoperative safety while also upgrading surgical training programs using automated skills evaluation tools and intraoperative feedback delivery [7].

In critical care settings, machine learning (ML) models have demonstrated superiority in predicting mortality and providing risk stratification, while unsupervised ML‐based phenotypic clustering has emerged as a strategy to connect clinical aspects to basic pathophysiology and treatment response [8]. While AI adoption is still in its preliminary stages in many societies [9], AI applications show particular promise in several areas like screening programs with considerably specific and sensitive autonomous models for disease detection, such as glaucoma [10], and in orthodontics for skeletal relationship analysis, facial attractiveness assessment, postpuberty mandibular growth prediction, and treatment progress supervision [11]. What may be most impressive is AI's ability to detect different medical image characteristics not promptly detectable by human beings—a retinal scan can provide information about blood pressure, glucose control, risk of Parkinson's, Alzheimer's, kidney, and hepatobiliary diseases, and the possibility of heart attack and stroke [4]. A review of the available literature shows that artificial intelligence has a significant impact on various medical fields and healthcare [12], even though some restrictions need consideration before applying deep‐learning techniques in general medical practice [13]. Nevertheless, these innovative tools have revolutionized the world, facilitating and enriching the work of medical professionals, ensuring higher precision, and providing a more personalized approach to patient care [11, 14, 15, 16, 17, 18].

AI integration into medical education presents distinct challenges, particularly regarding clinical reasoning processes. Given the nonlinear characteristics of deep learning, there is often no explanation of how AI systems arrive at their predictions, posing a significant challenge for medical education, where clinical reasoning forms the foundation of professional development [19]. Medical students face specific barriers, including limited formal AI education and significant concerns about the impact on healthcare delivery. Studies show that while students recognize AI as potentially beneficial for facilitating physician access to information (85.8%) and patient healthcare access (76.7%), many worry about potential reductions in physician services (44.9%) and devaluation of the medical profession (58.6%) [20].

Technical competency gaps represent another significant barrier, with only 6.0% of students reporting sufficient competence to make patients aware of AI characteristics and risks. Educational needs are particularly pronounced, with 96.2% of students demanding enhanced knowledge and skills associated with AI applications, 95.8% requiring training in AI‐based error reduction, and 93.8% needing education in managing AI‐related ethical challenges [20]. The disconnect between students' engagement with AI technology and formal AI education in medical curricula creates additional challenges in developing appropriate AI literacy [21].

This understanding of barriers is crucial because curriculum developers and instructors, upon awareness of students' readiness for the basic concepts of a topic, can plan the educational content presentation according to learners' requirements. Therefore, assessing readiness to understand knowledge and perspectives toward medical AI among students in this field seems vital for guiding instructional design and various developmental procedures, including curriculum development, pedagogical design, and needs analysis [1, 22, 23].

Additionally, some studies across various settings and contexts have examined medical students' readiness for, knowledge of, and attitudes toward artificial intelligence. Park (2021) assessed this population's perspectives on AI in medicine and showed high agreement among students (over 75%) regarding the significant contribution of AI to the future of the medical field, emphasizing the necessity of formal education on AI [24]. Similarly, Yüzbaşıoğlu (2021) examined dental students' knowledge of and attitudes toward AI and its potential dental applications, revealing the students' tendency to enhance their knowledge of artificial intelligence in dentistry [25]. Gray et al. (2022) identified major barriers, including lack of governmental structure and mechanism, resource limitation, and resistance to cultural change in curriculum and educational materials to AI application in medical practice [26].

Given the critical role of assessing medical students' preparedness to utilize AI technologies in medicine to outline subsequent steps for teaching artificial intelligence, examining this readiness seems essential. The Medical AI Readiness Scale for Medical Students (MAIRS‐MS), designed by Karaca et al. (2021), is a valid instrument for such assessments [27]. This scale is the only validated and dependable tool specifically designed to assess medical students' AI preparedness, and more importantly, it is the only tool translated to Persian with demonstrated validity and reliability [28].

Tung et al. (2023) assessed medical students' AI perspectives in Malaysia and evaluated their preparedness to use AI medical technologies through MAIRS‐MS. While most Malaysian students believed that artificial intelligence would play a major role in healthcare, they showed low readiness for AI adoption [29]. Aboalshamat et al. (2022) used MAIRS‐MS to evaluate artificial intelligence readiness among physicians and dentists in Saudi Arabia. Their results demonstrated that participants had low readiness to adopt artificial intelligence [30].

Studies assessing the current status of artificial intelligence in Iranian medical education demonstrate varying adoption patterns. A cross‐sectional survey conducted in Iranian medical institutions reported that medical students showed higher utilization rates of AI‐based decision support systems (89.5%) compared to physicians (45.1%), while both indicated frequent AI errors in their practice (physicians: 65.1%, students: 68.5%) [31]. The influence of experience on AI attitudes has been well‐documented, with less experienced practitioners showing more favorable perspectives toward AI implementation (*p* = 0.02) [31]. Studies conducted at Smart University of Medical Sciences indicate that while structured AI education programs are being implemented, there remains a need for more comprehensive training to address both technical competencies and practical applications in medical education [32]. While several international studies have assessed AI readiness in medical education, scant research has specifically examined Iranian medical students' preparedness. Thus, this paper investigates medical students' readiness at Kerman University of Medical Sciences (KMU) to adopt AI in medicine in 2022, addressing this gap in the literature and seeking to enhance the increasingly growing international research on medical AI education.

## 2 Methods

### 2.1 Research Design and Setting

This cross‐sectional research was carried out from November to December 2022 at KMU.

### 2.2 Ethical Considerations

The research ethics were approved by the KMU institutional review board (IR.KMU.AH.REC.1401.253). Eligible participants were identified through university registration lists and initially contacted via text messages. Study information links were then shared through online messaging platforms. The study information, including objectives and data handling procedures, was provided to all participants. Electronic informed consent was obtained through the online survey platform before the questionnaire was presented. Data were collected anonymously and saved securely with access restricted to the research team only.

### 2.3 Study Participants and Sampling

The population consisted of KMU medical students in 2022. Through simple random sampling, students were contacted using the university registration system and their registered phone numbers. Cochran's formula for finite populations was utilized to determine the sample size. With a total medical student population of 1200, 95% confidence level (*Z* = 1.96), 5% margin of error, and *p*‐value of 0.5, the calculated minimum sample size was 291 participants (*n* = (1200 × 1.96² × 0.5 × 0.5)/(0.05² × 1199 + 1.96² × 0.5 × 0.5) = 291), which was increased to 360 to account for potential non‐responses. The inclusion criteria were studying medicine and willingness to participate in research. Questionnaires with >10% unanswered questions were excluded.

### 2.4 Data Collection Tools and Techniques

The MAIRS‐MS has been previously translated into Persian and psychometrically validated by Rezazadeh et al. (2023). Cronbach's alpha was employed to assess the scale's reliability, revealing an acceptable value of 0.94. Content validity index (0.92) and content validity ratio (0.75) were used for the content validity measurements. All hypothesized factors showed adequate fit according to confirmatory factor analysis.

The Persian MAIRS‐MS version contains two parts, with the first covering demographic data such as gender and level of education. The second part outlines 20 items in four domains of “cognition” (8 items), “ability” (7 items), “vision” (2 items), and “ethics” (3 items). Participants' attitudes are examined on a 5‐point Likert scale from 1 to 5, representing strongly disagree to strongly agree, respectively [28].

The cognition domain measures the student's terminological knowledge of medical AI usages, their logics, and data science. The second domain assesses the student's competencies in selecting suitable medical AI tools, properly integrating them with professional knowledge, and effectively conveying AI insights. The vision domain determines students' abilities to articulate restrictions, strengths, and drawbacks associated with medical AI, predict potential benefits and risks, and envision innovations. The last domain refers to the student's commitment to legal and ethical principles and regulations throughout healthcare AI deployment [27].

### 2.5 Data Collection and Analysis

After introducing the study objectives and necessity, the students were invited to collaborate, and their confidence in their participation was built to minimize incomplete and erroneous data. The online MAIRS‐MS was distributed through online messaging platforms. Non‐responders were followed up after 2 weeks using the same online messaging platforms. Responses were automatically captured through the online survey platform to eliminate manual entry errors and ensure data quality. A research methodologist and a biostatistician reviewed the data independently for completeness and consistency. Any discrepancies were resolved through discussion with the principal researcher.

### 2.6 Data Analysis

The collected data were processed and analyzed by SPSS 24.0. Descriptive statistical techniques were primarily used. Independent sample *t*‐tests helped evaluate differences in mean questionnaire scores across variables. Statistical significance was represented by *p* < 0.05.

## 3 Results

Of 360 distributed questionnaires, 340 were completed and subsequently included in the study (response rate = 94%). The demographic characteristics of medical students showed that of 340 participants, 265 (77.9%) were female and 75 (22.1%) were male. Regarding their educational level, 197 (58%) were basic sciences students, 112 (33%) were in their clinical clerkship phase, and 31 (9%) were interns. The participants' enrollment years ranged from 2017 to 2022, with the majority entering medical school in 2021 (27.06%) and 2020 (26.18%).

**FIGURE 1:**
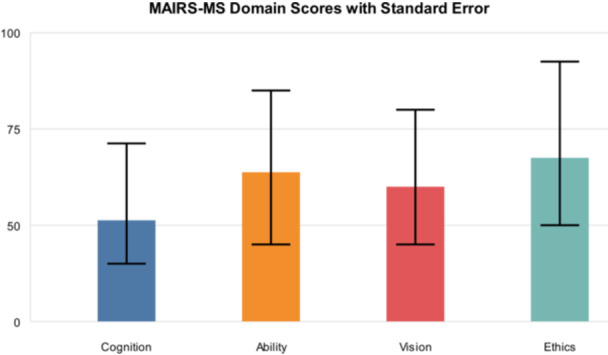
Distribution of MAIRS‐MS domain scores with standard errors, showing the percentage of maximum possible scores for each domain. MAIRS‐MS, Medical Artificial Intelligence Readiness Scale for Medical Students.

The highest score was related to the “ability” domain (*M* = 21.88, SD = 6.74), followed by “cognition” (*M* = 20.30, SD = 7.04), “ethics” (*M* = 10.94, SD = 3.04), and “vision” (*M* = 6.09, SD = 1.94), respectively. Analysis of AI readiness scores across different domains revealed varying levels of preparedness, with respective score ranges of 8–40, 7–35, 2–10, 3–15, and 20–100 for cognition, ability, vision, ethics, and total score, relatively. Table 1 and Figure 1 present the analysis results for the questionnaire domains.

**TABLE 1:** Results of the analysis of the MAIRS‐MS domains (*N* = 340).

Domain	Mean ± SD.	% of Maximum Score*	95% CI	Range
Cognition	20.30 ± 7.04	50.75%	[19.53, 21.07]	8–40
Ability	21.88 ± 6.74	62.51%	[21.14, 22.62]	7–35
Vision	6.09 ± 1.94	60.90%	[5.88, 6.30]	2–10
Ethics	10.94 ± 3.04	72.93%	[10.61, 11.27]	3–15
Total	59.21 ± 16.12	59.21%	[57.46, 60.96]	20–100

Abbreviation: MAIRS‐MS, Medical Artificial Intelligence Readiness Scale for Medical Students.

* Percentage of the maximum possible score for each domain.

**FIGURE 2:**
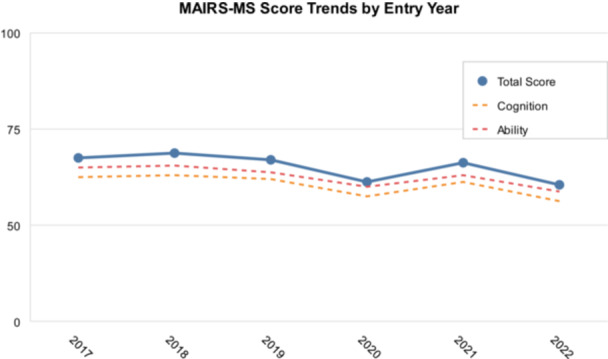
Trends in MAIRS‐MS scores by entry year (2017–2022) showing total scores and domain‐specific patterns. A slight downward trend is observed in more recent academic years. MAIRS‐MS, Medical Artificial Intelligence Readiness Scale for Medical Students.

Further analysis of scores across different academic years revealed variations in readiness levels. As shown in Table 2 and Figure 2, there was a slight downward trend in overall readiness scores from earlier to more recent academic years, with the highest mean total score observed in the 2018 cohort (62.91 ± 19.31) and the lowest in the 2022 cohort (56.63 ± 16.59)

**TABLE 2:** MAIRS‐MS scores by academic year.

Entry year	*N* (%)	Total score (Mean ± SD)	Cognition	Ability	Vision	Ethics
2017	25 (7.35%)	61.28 ± 12.94	21.04	23.16	6.48	10.60
2018	32 (9.41%)	62.91 ± 19.31	21.44	23.53	6.47	11.47
2019	48 (14.12%)	60.92 ± 12.93	20.73	22.44	6.25	11.50
2020	54 (15.88%)	57.17 ± 18.23	19.43	21.06	5.85	10.83
2021	89 (26.18%)	60.24 ± 15.23	20.56	22.11	6.13	11.44
2022	92 (27.06%)	56.63 ± 16.59	19.29	20.78	5.87	10.69

Abbreviation: MAIRS‐MS, Medical Artificial Intelligence Readiness Scale for Medical Students.

**FIGURE 3:**
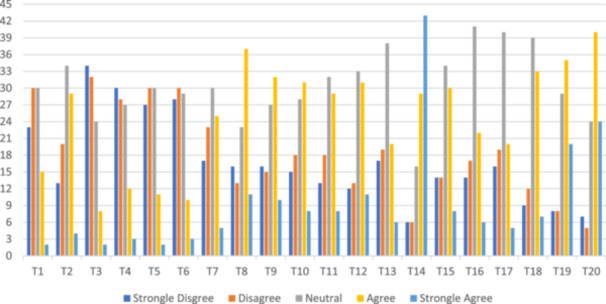
Students' responses to MAIRS‐MS (Percentage/Item's ID). MAIRS‐MS, Medical Artificial Intelligence Readiness Scale for Medical Students.

The highest score was related to the item “I find it valuable to use AI for education, service, and research purposes.” (42.6% strongly agree, mean = 3.96, standard deviation=1.18). The lowest score was related to the item “I can explain how AI systems are trained.” (1.5% strongly agree, mean = 2.10, standard deviation = 1.01). These results clearly demonstrate the need to design and deliver educational programs on AI applications in medicine for students. Table 3 and Figure 3 present the students' responses to MAIRS‐MS.

**TABLE 3:** The students' responses to MAIRS‐MS.

ID	Item	Mean	Standard deviation
T1	I can define the basic concepts of data science	2.43	1.07
T2	I can define the basic concepts of statistics	2.92	1.07
T3	I can explain how AI systems are trained	2.10	1.01
T4	I can define the basic concepts and terminology of AI	2.31	1.11
T5	I can properly analyze the data obtained by AI in healthcare	2.32	1.05
T6	I can differentiate the functions and features of AI‐related tools and applications	2.30	1.07
T7	I can organize workflows compatible with AI	2.77	1.14
T8	I can express the importance of data collection, analysis, evaluation, and safety; for the development of AI in healthcare	3.16	1.25
T9	I can harness AI‐based information combined with my professional knowledge	3.06	1.23
T10	I can use AI technologies effectively and efficiently in healthcare delivery	3.01	1.19
T11	I can use artificial intelligence applications in accordance with its purpose	3.01	1.15
T12	I can access, evaluate, use, share and create new knowledge using information and communication technologies	3.15	1.15
T13	I can explain how AI applications offer a solution to which problem in healthcare	2.79	1.11
T14	I find valuable to use AI for education, service, and research purposes	3.96	1.18
T15	I can choose proper AI application for the problem encountered in healthcare	2.90	1.09
T16	I can explain the limitations of AI technology	2.92	1.06
T17	I can foresee the opportunities and threats that AI technology can create	3.17	1.03
T18	I can use health data in accordance with legal and ethical norms	3.51	1.13
T19	I can conduct under ethical principles while using AI technologies	3.74	1.05
T20	I can follow legal regulations regarding the use of AI technologies in healthcare	3.69	1.10
	Total	59.21	16.12

Abbreviation: MAIRS‐MS, Medical Artificial Intelligence Readiness Scale for Medical Students.

We looked for associations between the demographic data and the four domains of the questionnaire. The Wilcoxon test showed no significant correlations between gender and the AI scores (*p* = 0.61). Similarly, using the Kruskal‐Wallis test and considering the relationship between AI scores and education level, no significant correlations were found between AI scores and education level (*p* = 0.44). Additionally, enrollment year had no significant correlations with AI scores (*p* > 0.05). Overall, the demographic characteristics did not have any significant correlations with the AI scores obtained (*p* > 0.05).

According to the Correlation analysis in Table 4, all domains revealed significant positive correlations (*p* < 0.001). The strongest correlation was found between Ability and Vision (*r* = 0.806), while Ethics and Cognition showed the weakest correlation (*r* = 0.441). These patterns suggest that while AI competencies are interconnected, technical knowledge may not necessarily align with ethical awareness.

**TABLE 4:** Correlations between MAIRS‐MS domains (*N* = 340).

Domain	Cognition	Ability	Vision	Ethics
Cognition	1	0.702	0.712	0.441
Ability	0.702	1	0.806	0.707
Vision	0.712	0.806	1	0.596
Ethics	0.441	0.707	0.596	1

*Note:* Significant correlation at the 0.01 level (two‐tailed).

Abbreviation: MAIRS‐MS, Medical Artificial Intelligence Readiness Scale for Medical Students.

Table 5 comparatively analyzes MAIRS‐MS scores across different countries, providing an international context for our findings and highlighting the differences in medical students' readiness for AI technologies. The table includes data from Iran, where the current study involved 340 participants, revealing a high score in the ethics domain (72.93%) but lower scores in cognition (50.75%) and ability (62.51%). Similar trends were observed in Saudi Arabia, with a larger sample size of 572 and only 14.5% of students receiving formal AI training. In the same vein, Jordanian students showed a correlation between academic performance and AI readiness. Malaysian studies demonstrated significant correlations between age and academic year with readiness levels, while Lebanese students expressed a high interest in AI learning despite lower scores. Qatari students expressed a strong demand for AI knowledge in healthcare, whereas Indian students emphasized the need for focused educational programs. Turkish studies revealed varying levels of awareness and readiness, with one study indicating high AI anxiety. Lastly, Indonesian students showed a positive correlation between AI readiness and prior coding experience. Overall, the table illustrates the diverse landscape of AI readiness among medical students across various cultural and educational contexts.

**TABLE 5:** International comparison of MAIRS‐MS domain scores across different countries.

Country	Sample size	Ethics (%)	Cognition (%)	Ability (%)	Vision (%)	Key findings	Ref
Iran (current study)	340	72.93	50.75	62.51	60.90	No significant demographic correlations; highest scores in ethics domain	—
Saudi Arabia	572	70.7	56.7	65.3	59.8	Only 14.5% received formal AI training; computer skills correlated with scores	[33]
Jordan	858	61.9	56.4	58.5	60.1	Academic performance correlated with AI readiness	[34]
Malaysia (Study 1)	105	67.1	69.0	67.9	67.9	Significant correlation between age, academic year, and readiness	[35]
Malaysia (Study 2)	301	73.3	52.5	62.5	66.7	87.36% believe AI will play an important role in healthcare	[29]
Lebanon	365	56.7	46.0	32.9	57.0	High interest in AI learning despite low scores	[36]
Qatar	94	68.6	60.6	66.2	70.0	High demand (84%) for AI healthcare knowledge	[37]
India	482	69.3	65.6	69.1	69.1	Need for focused educational programs	[38]
Turkey (Study 1)	172	68.7	57.5	68.3	65.0	High awareness of medical AI applications	[39]
Turkey (Study 2)	542	67.0	46.4	59.5	57.2	Moderate readiness levels; high AI anxiety	[40]
Indonesia	650	61.3	69.5	70.5	69.8	Positive correlation with prior coding experience	[41]

*Note:* Percentages represent the proportion of the maximum possible score in each domain.

Abbreviation: MAIRS‐MS, Medical Artificial Intelligence Readiness Scale for Medical Students.

## 4 Discussion

This study provides some early insights into medical students' perceptions of AI readiness and applications in the medical field by evaluating the contributions of four key aspects: cognition, ability, vision, and ethics. The research has employed the MAIRS‐MS for the first time to investigate Iranian medical students' preparedness for AI adoption.

Our study evaluated AI readiness among medical students, seeking to address a gap in previous research with a primary focus on knowledge and attitudes [30, 42, 43]. Using the validated MAIRS‐MS instrument, the findings revealed scores of 2.10 to 3.96 on a 1 to 5 scale for most participants, which was below the midpoint of 3 and indicated low AI readiness levels.

This finding aligns with international patterns, as shown in Table 5, where varying readiness levels are observed across different countries. While Turkish medical students showed similar overall readiness levels (59.5%) to our findings (59.21%), Malaysian students demonstrated higher scores across all domains (67.9%) [35]. Studies from Saudi Arabia and Jordan reported comparable challenges, particularly regarding formal AI education [33, 34].

Aboalshamat et al. (2022) evaluated AI readiness among medical and dental specialists and students in Saudi Arabia using the MAIRS‐MS, indicating their low AI readiness levels. Their results highlighted the need for the inclusion of AI and its practical education at the undergraduate level [30].

One unexpected finding was the relatively high performance in the ethics domain (72.93%) compared to other domains, aligning with similar studies in Saudi Arabia (70.7%) [33], Malaysia (67.1%–73.3%) [29, 35], and Turkey (67%–68.7%) [39, 40]. This consistency across different cultural contexts suggests that medical students generally demonstrate strong ethical awareness regarding AI applications, possibly due to the emphasis on medical ethics in traditional curricula.

Another notable finding was a lack of significant demographic correlations with AI readiness scores in our study (*p* > 0.05). This contrasts with findings from Malaysia, where significant relationships were found between age and academic year with AI readiness scores, and Indonesia, where gender and prior coding experience showed positive correlations with readiness levels [41]. The absence of such correlations in the present study might reflect the uniformity of AI exposure across different student groups in Iran.

We found that the items in the “ability” domain showed the most important aspects of medical students' AI readiness and tendency toward its adoption in the medical field. These items highlighted competencies in selecting appropriate medical AI applications, properly combining them with professional knowledge, and explaining them to patients. Interestingly, our findings showed lower ability scores (62.51%) compared to several countries, including Malaysia (67.9%) [35] and Indonesia (70.5%) [41], suggesting specific challenges in practical AI application skills among Iranian students. Sadoughi et al. (2018) reviewed various AI‐based image processing methods for breast cancer diagnosis and demonstrated that computerized breast cancer diagnosis involves advanced medicine and is clearly acceptable ethically and medically, considering its impacts [44]. Alowais et al. (2023) highlighted the capacity of AI to diagnose diseases, develop personalized treatment plans, and support clinician decision‐making [45]. Both studies recommended physician training, especially during student years, as a worthwhile investment to enhance diagnosis approaches.

The “cognition” domain concerns students' cognitive preparedness regarding medical AI applications’ terminological knowledge, artificial intelligence, and data science logic. Our students' cognition scores (50.75%) were notably lower than those reported in most other countries (Table 5), with only Lebanon reporting lower scores (46.0%) [36]. Our findings align with Markus et al. (2021), who identified a lack of transparency as a primary barrier to AI implementation in healthcare, highlighting the potential of AI as a strategy to address these concerns and constituting a step toward trustworthy AI [46].

The MAIRS‐MS “ethics” domain measures commitment to pertinent legal and ethical guidelines when applying AI in healthcare. Despite achieving the highest domain score among our participants, several challenges remain in translating ethical awareness into practice. Wang et al. (2023) highlighted that healthcare AI must follow strict ethical principles, reassuring healthcare experts about the responsible application of AI, promoting reliable information exchange, safeguarding patient privacy, and enabling patients to make informed treatment decisions [47]. Zhang et al. (2023) considered the ethical issues in medical AI and encouraged further research to investigate AI risks and social impacts while strengthening international collaboration [48].

As indicated, the “vision” domain items reflected the lowest aspects of medical students' readiness for AI adoption in the medical field. Our vision domain scores (60.90%) were lower than those reported in most Asian countries (Table 4), particularly compared to Qatar (70.0%) [37] and India (69.1%) [38]. This domain refers to individuals' ability to articulate limitations, strengths, and weaknesses, predict opportunities and threats, and formulate ideas regarding medical AI. Gödde et al. (2023) reviewed the strengths, drawbacks, chances, and threats of ChatGPT in the medical literature, indicating that in spite of clear opportunities, some guidelines must be followed to ensure strengths in clinical practice and protect against threats. They emphasized the necessity of providing informal AI training in the educational programs of medicine [49]. Noguerol et al. (2019) explained the weakness of AI‐based approaches in radiology, including not being able to interpret the patient's clinical context and the need to ensure data privacy with correct reliable data labeling [50].

Several factors appear to contribute to low readiness levels observed in our study. First, as seen across multiple countries (Table 5), limited formal AI education represents a significant barrier. Second, data from Saudi Arabia suggests that proficiency in English and computer skills significantly influences AI readiness—factors that may also affect Iranian students but weren't directly measured in our study [33]. Third, findings from Turkish studies indicate that AI anxiety may impact readiness levels, with higher anxiety correlating with lower readiness scores.

Our findings demonstrate the necessity of integrating formal or informal AI education into medical programs. In this regard, Xuan et al. (2022) utilized the MAIRS‐MS in a Malaysian study to evaluate medical students' AI readiness. They recommended that education policymakers introduce more AI educational programs, especially introductory courses for undergraduates, to enable future enhanced confidence in interacting with AI technologies [51]. Liu et al. (2022) revealed that medical students recognize AI's importance, yet current formal education programs and learning resources on AI topics are limited in most American medical schools [52]. These studies indicate that both developed and developing countries have restricted medical AI curricula, necessitating undergraduate educational programs to enhance competencies, as future physicians will undoubtedly leverage AI in daily practice.

To expand future research directions, we recommend longitudinal studies that track AI readiness over time and assess the influence of integrated AI curricula on students' competencies and confidence in using AI technologies.

Incorporating a discussion of cultural and educational contexts is essential for a comprehensive understanding of medical students' readiness for AI technologies. Cultural factors, such as societal attitudes toward technology, educational practices, and the emphasis on ethical considerations, significantly influence how students perceive and engage with AI in medicine. For instance, in cultures prioritizing technological innovation and integration, students may have a more favorable view of AI and its applications in healthcare. This can lead to higher readiness levels, as seen in countries like Malaysia and Qatar, where there is a strong demand for AI knowledge in healthcare. Conversely, in cultures dominated by skepticism about technology or limited AI exposure, students may exhibit lower readiness levels, as observed in Lebanon. Additionally, the educational context plays a critical role. Countries with robust AI curricula and hands‐on training opportunities tend to produce graduates with higher readiness for AI adoption in their medical practice. In contrast, regions lacking formal AI education may result in students feeling unprepared, even if they have a theoretical understanding of the subject. By examining these cultural and educational dynamics, we can gain deeper insights into the variations in readiness levels across different countries, subsequently informing the advancement of targeted educational programs and initiatives to address certain cultural concerns and promote AI integration into medical education globally.

To integrate AI into medical curricula, we propose a step‐by‐step guide that includes the following stages: first, a Needs Assessment is conducted to identify gaps in current curricula related to AI. Next, collaboration with AI experts is essential for designing relevant courses in the Curriculum Development phase. Following this, Pilot Programs are implemented to gather feedback. Finally, the effectiveness of the programs is continuously assessed, and necessary adjustments are made in the Evaluation and Revision stage.

We suggest outlining implementation strategies and resource requirements for effective AI curriculum integration. This involves Training Faculty by investing in training programs for educators to ensure they are well‐equipped to teach AI concepts. Additionally, Resource Allocation is crucial, requiring the allocation of resources for the technological tools and materials needed for AI training. Furthermore, Collaboration with Industry is essential, as partnering with AI companies can provide real‐world insights and resources that enhance the learning experience.

Several limitations should be considered when interpreting our findings. Beyond the inherent limitations of self‐report questionnaires, single‐institution sampling, and convenience sampling methodology, we notably did not collect data on participants' previous AI exposure or English language proficiency—factors that other studies have identified as significant predictors of AI readiness. This is the first assessment of Iranian medical students' AI preparedness and could serve as a benchmark for future research. A key strength is the application of the validated MAIRS‐MS to gauge preparedness. We suggest further studies in educational settings for more meaningful cross‐cultural comparisons.

## 5 Conclusion

AI is advancing globally across all domains, including medicine, increasingly contributing to humans' daily lives, which emphasizes the need for extensive AI education. As highlighted by the low readiness scores, medical students have not gained AI readiness yet. Therefore, it is recommended that medical science universities include AI educational programs in undergraduate and postgraduate contexts to ensure that the next generation of medical professionals fit for the near AI ecosystem.

## Author Contributions

**Hossein Rezazadeh:** writing – original draft, writing – review and editing, project administration, supervision. **Ali Madadi Mahani:** writing – original draft, writing – review and editing. **Mahla Salajegheh:** conceptualization, writing – original draft, writing – review and editing. All authors have read and approved the final version of the manuscript.

## Declaration of Generative AI and AI‐Assisted Technologies in the Writing Process

The authors utilized the AI assistant Claude to paraphrase select passages during the drafting process. After generating paraphrased versions, the authors thoroughly reviewed the output, editing and revising the language as necessary. The authors take full accountability for the final published content.

## Ethics Statement

The KMU's institutional review board approved this study (No. IR.KMU.AH.REC.1401.253). The participants did not receive any incentives, and participation was voluntary. Verbal and written consent for participation was obtained based on the proposal approved by the ethics committee. The participants were also assured of the confidentiality of their information, and it was explained that the results would only be used for research objectives.

## Consent

The authors have nothing to report.

## Conflicts of Interest

The authors declare no conflicts of interest.

## Transparency Statement

The lead author, Mahla Salajegheh, affirms that this manuscript is an honest, accurate, and transparent account of the study being reported; that no important aspects of the study have been omitted; and that any discrepancies from the study as planned (and, if relevant, registered) have been explained.

## Data Availability

The authors confirm that the data supporting the findings of this study are available within the article. The raw datasets generated during the current study are available from the corresponding author upon reasonable request with appropriate ethics approval and data sharing agreement. No additional external data were used or generated. Mahla Salajegheh had full access to all of the data in this study and takes complete responsibility for the integrity of the data and the accuracy of the data analysis.

## References

[1] C. Sit , R. Srinivasan , A. Amlani , et al., “Attitudes and Perceptions of UK Medical Students Towards Artificial Intelligence and Radiology: A Multicentre Survey,” Insights into Imaging 11, no. 1 (2020): 14. 32025951 10.1186/s13244-019-0830-7 PMC7002761

[2] Q. Sun , A. Akman , and B. W. Schuller , “Explainable Artificial Intelligence for Medical Applications: A Review,” ACM Transactions on Computing for Healthcare 6 (2024): 17.

[3] K. R. Siegersma , T. Leiner , D. P. Chew , Y. Appelman , L. Hofstra , and J. W. Verjans , “Artificial Intelligence in Cardiovascular Imaging: State of the Art and Implications for the Imaging Cardiologist,” Netherlands Heart Journal 27, no. 9 (2019): 403–413. 31399886 10.1007/s12471-019-01311-1 PMC6712136

[4] E. J. Topol , “As Artificial Intelligence Goes Multimodal, Medical Applications Multiply,” Science 381, no. 6663 (2023): eadk6139. 10.1126/science.adk6139 37708283

[5] G. Bedi , F. Carrillo , G. A. Cecchi , et al., “Automated Analysis of Free Speech Predicts Psychosis Onset in High‐Risk Youths,” NPJ Schizophrenia 1, no. 1 (2015): 15030. 27336038 10.1038/npjschz.2015.30 PMC4849456

[6] H.‐Y. Jin , M. Zhang , and B. Hu , “Techniques to Integrate Artificial Intelligence Systems With Medical Information in Gastroenterology,” Artificial Intelligence in Gastrointestinal Endoscopy 1, no. 1 (2020): 19–27.

[7] J. E. Knudsen , U. Ghaffar , R. Ma , and A. J. Hung , “Clinical Applications of Artificial Intelligence in Robotic Surgery,” Journal of Robotic Surgery 18, no. 1 (2024): 102. 38427094 10.1007/s11701-024-01867-0 PMC10907451

[8] J. C. Jentzer , A. H. Kashou , and D. H. Murphree , “Clinical Applications of Artificial Intelligence and Machine Learning in the Modern Cardiac Intensive Care Unit,” Intelligence‐Based Medicine 7 (2023): 100089.

[9] A. S. Ahuja , “The Impact of Artificial Intelligence in Medicine on the Future Role of the Physician,” PeerJ 7 (2019): e7702. 31592346 10.7717/peerj.7702 PMC6779111

[10] S. Yousefi , “Clinical Applications of Artificial Intelligence in Glaucoma,” Journal of Ophthalmic & Vision Research 18, no. 1 (2023): 97–112. 36937202 10.18502/jovr.v18i1.12730 PMC10020779

[11] G. Dipalma , A. D. Inchingolo , A. M. Inchingolo , et al., “Artificial Intelligence and Its Clinical Applications in Orthodontics: A Systematic Review,” Diagnostics 13, no. 24 (2023): 3677. 38132261 10.3390/diagnostics13243677 PMC10743240

[12] B. Mesko , “The Role of Artificial Intelligence in Precision Medicine,” Expert Review of Precision Medicine and Drug Development 2, no. 5 (September 2017): 239–241.

[13] N. Fujima , K. Kamagata , D. Ueda , et al., “Current State of Artificial Intelligence in Clinical Applications for Head and Neck MR Imaging,” Magnetic Resonance in Medical Sciences 22, no. 4 (2023): 401–414. 37532584 10.2463/mrms.rev.2023-0047 PMC10552661

[14] A. Holzinger , G. Langs , H. Denk , K. Zatloukal , and H. Müller , “Causability and Explainability of Artificial Intelligence in Medicine,” WIREs Data Mining and Knowledge Discovery 9, no. 4 (2019): e1312. 10.1002/widm.1312 PMC7017860 32089788

[15] S. Azzi , S. Gagnon , A. Ramirez , and G. Richards , “Healthcare Applications of Artificial Intelligence and Analytics: A Review and Proposed Framework,” Applied Sciences 10, no. 18 (2020): 6553.

[16] A. Ramesh , C. Kambhampati , J. Monson , and P. Drew , “Artificial Intelligence in Medicine,” Annals of the Royal College of Surgeons of England 86 (2004): 334–338. 15333167 10.1308/147870804290 PMC1964229

[17] F. Shi , J. Wang , J. Shi , et al., “Review of Artificial Intelligence Techniques in Imaging Data Acquisition, Segmentation, and Diagnosis for COVID‐19,” IEEE Reviews in Biomedical Engineering 14 (2021): 4–15. 32305937 10.1109/RBME.2020.2987975

[18] P. Hamet and J. Tremblay , “Artificial Intelligence in Medicine,” Metabolism: Clinical and Experimental 69 (2017): S36–S40. 10.1016/j.metabol.2017.01.011 28126242

[19] K. S. Chan and N. Zary , “Applications and Challenges of Implementing Artificial Intelligence in Medical Education: Integrative Review,” JMIR Medical Education 5, no. 1 (2019): e13930. 31199295 10.2196/13930 PMC6598417

[20] M. M. Civaner , Y. Uncu , F. Bulut , E. G. Chalil , and A. Tatli , “Artificial Intelligence in Medical Education: A Cross‐Sectional Needs Assessment,” BMC Medical Education 22, no. 1 (2022): 772. 36352431 10.1186/s12909-022-03852-3 PMC9646274

[21] L. Weidener and M. Fischer , “Artificial Intelligence In Medicine: Cross‐Sectional Study Among Medical Students on Application, Education, and Ethical Aspects,” JMIR Medical Education 10, no. 1 (2024): e51247. 38180787 10.2196/51247 PMC10799276

[22] B. Gong , J. P. Nugent , W. Guest , et al., “Influence of Artificial Intelligence on Canadian Medical Students' Preference for Radiology Specialty: Anational Survey Study,” Academic Radiology 26, no. 4 (2019): 566–577. 30424998 10.1016/j.acra.2018.10.007

[23] D. Pinto Dos Santos , D. Giese , S. Brodehl , et al., “Medical Students' Attitude Towards Artificial Intelligence: A Multicentre Survey,” European Radiology 29, no. 4 (2019): 1640–1646. 29980928 10.1007/s00330-018-5601-1

[24] C. J. Park , P. H. Yi , and E. L. Siegel , “Medical Student Perspectives on the Impact of Artificial Intelligence on the Practice of Medicine,” Current Problems in Diagnostic Radiology 50, no. 5 (2021): 614–619. 32680632 10.1067/j.cpradiol.2020.06.011

[25] E. Yüzbaşıoğlu , “Attitudes and Perceptions of Dental Students Towards Artificial Intelligence,” Journal of Dental Education 85, no. 1 (2021): 60–68. 32851649 10.1002/jdd.12385

[26] K. Gray , J. Slavotinek , G. L. Dimaguila , and D. Choo , “Artificial Intelligence Education for the Health Workforce: Expert Survey of Approaches and Needs,” JMIR Medical Education 8, no. 2 (2022): e35223. 35249885 10.2196/35223 PMC9016514

[27] O. Karaca , S. A. Çalışkan , and K. Demir , “Medical Artificial Intelligence Readiness Scale for Medical Students (MAIRS‐MS) – Development, Validity and Reliability Study,” BMC Medical Education 21, no. 1 (2021): 112. 33602196 10.1186/s12909-021-02546-6 PMC7890640

[28] H. Rezazadeh , H. Ahmadipour , and M. Salajegheh , “Psychometric Evaluation of Persian Version of Medical Artificial Intelligence Readiness Scale for Medical Students,” BMC Medical Education 23, no. 1 (2023): 527. 37488522 10.1186/s12909-023-04516-6 PMC10367280

[29] A. Y. Z. Tung and L. W. Dong , “Malaysian Medical Students' Attitudes and Readiness Toward AI (Artificial Intelligence): A Cross‐Sectional Study,” Journal of Medical Education and Curricular Development 10 (2023): 23821205231201164. 37719325 10.1177/23821205231201164 PMC10501060

[30] K. Aboalshamat , R. Alhuzali , A. Alalyani , et al., “Medical and Dental Professionals Readiness for Artificial Intelligence for Saudi Arabia Vision 2030,” International Journal of Pharmaceutical Research and Allied Sciences 11, no. 4 (2022): 52–59.

[31] E. Esfandiari , F. Kalroozi , N. Mehrabi , and Y. Hosseini , “Knowledge and Acceptance of Artificial Intelligence and its Applications Among the Physicians Working in Military Medical Centers Affiliated With Aja University: A Cross‐Sectional Study,” Journal of Education and Health Promotion 13, no. 1 (July 2024): 271. 39309999 10.4103/jehp.jehp_898_23 PMC11414869

[32] B. Sabet , H. Khani , A. Namaki , A. Habibi , S. Rajabzadeh , and S. Shafiekhani , “Evaluation of Artificial Intelligence Fall School Program at Smart University of Medical Sciences,” Research and Development in Medical Education 12, no. 1 (2023): 23.

[33] A. Al Shahrani , N. Alhumaidan , Z. AlHindawi , et al., “Readiness to Embrace Artificial Intelligence Among Medical Students in Saudi Arabia: A National Survey,” Healthcare 12 (2024): 2504. 39765931 10.3390/healthcare12242504 PMC11727990

[34] M. Hamad , F. Qtaishat , E. Mhairat , et al., “Artificial Intelligence Readiness Among Jordanian Medical Students: Using Medical Artificial Intelligence Readiness Scale For Medical Students (MAIRS‐MS),” Journal of medical education and curricular development 11 (2024): 23821205241281648. 39346121 10.1177/23821205241281648 PMC11437586

[35] P. Y. Xuan , M. I. F. Fahumida , M. I. Al Nazir Hussain , et al., “Readiness Towards Artificial Intelligence Among Undergraduate Medical Students in Malaysia,” Education in Medicine Journal 15, no. 2 (2023): 49–60.

[36] O. A. Daher , A. A. Dabbousi , R. Chamroukh , A. Y. Saab , A. R. Al Ayoubi , and P. Salameh , “Artificial Intelligence: Knowledge and Attitude Among Lebanese Medical Students,” Cureus 16, no. 1 (2024): e51466. 38298326 10.7759/cureus.51466 PMC10829838

[37] D. Hammoudi Halat , R. Shami , A. Daud , W. Sami , A. Soltani , and A. Malki , “Artificial Intelligence Readiness, Perceptions, and Educational Needs Among Dental Students: A Cross‐Sectional Study,” Clinical and Experimental Dental Research 10, no. 4 (2024): e925. 38970241 10.1002/cre2.925 PMC11226543

[38] D. Dhurandhar , M. Dhamande , S. C , P. Bhadoria , T. Chandrakar , and J. Agrawal , “Exploring Medical Artificial Intelligence Readiness Among Future Physicians: Insights From a Medical College in Central India,” Cureus 17, no. 1 (2025): e76835. 39897272 10.7759/cureus.76835 PMC11787952

[39] B. Emir , T. Yurdem , T. Ozel , et al., “Artificial Intelligence Readiness Status of Medical Faculty Students,” Konuralp Tıp Dergisi 16, no. 1 (2024): 88–95.

[40] G. Ö. Güven , Ş. Yilmaz , and F. Inceoğlu , “Determining Medical Students' Anxiety and Readiness Levels About Artificial Intelligence,” Heliyon 10, no. 4 (2024): e25894. 38384508 10.1016/j.heliyon.2024.e25894 PMC10878911

[41] N. P. H. Lugito , C. Cucunawangsih , N. Suryadinata , et al., “Readiness, Knowledge, and Perception Towards Artificial Intelligence of Medical Students at Faculty of Medicine, Pelita Harapan University, Indonesia: A Cross Sectional Study,” BMC Medical Education 24, no. 1 (2024): 1044. 39334022 10.1186/s12909-024-06058-x PMC11430330

[42] S. F. Mousavi Baigi , M. Sarbaz , K. Ghaddaripouri , M. Ghaddaripouri , A. S. Mousavi , and K. Kimiafar , “Attitudes, Knowledge, and Skills Towards Artificial Intelligence Among Healthcare Students: A Systematic Review,” Health Science Reports 6, no. 3 (2023): e1138. 36923372 10.1002/hsr2.1138 PMC10009305

[43] J. W. Ampofo , C. V. Emery , and I. N. Ofori , “Assessing the Level of Understanding (Knowledge) and Awareness of Diagnostic Imaging Students in Ghana on Artificial Intelligence and Its Applications in Medical Imaging,” Radiology Research and Practice 2023 (2023): 1–9. 10.1155/2023/4704342 PMC10287516 37362195

[44] F. Sadoughi , Z. Kazemy , F. Hamedan , L. Owji , M. Rahmanikatigari , and T. T. Azadboni , “Artificial Intelligence Methods for the Diagnosis of Breast Cancer by Image Processing: A Review,” Breast Cancer (Dove Medical Press) 10 (2018): 219–230. 30555254 10.2147/BCTT.S175311 PMC6278839

[45] S. A. Alowais , S. S. Alghamdi , N. Alsuhebany , et al., “Revolutionizing Healthcare: The Role of Artificial Intelligence in Clinical Practice,” BMC Medical Education 23, no. 1 (2023): 689. 37740191 10.1186/s12909-023-04698-z PMC10517477

[46] A. F. Markus , J. A. Kors , and P. R. Rijnbeek , “The Role of Explainability in Creating Trustworthy Artificial Intelligence for Health Care: A Comprehensive Survey of the Terminology, Design Choices, and Evaluation Strategies,” Journal of Biomedical Informatics 113 (2021): 103655. 33309898 10.1016/j.jbi.2020.103655

[47] C. Wang , S. Liu , H. Yang , J. Guo , Y. Wu , and J. Liu , “Ethical Considerations of Using ChatGPT in Health Care,” Journal of Medical Internet Research 25 (2023): e48009. 37566454 10.2196/48009 PMC10457697

[48] J. Zhang and Z. Zhang , “Ethics and Governance of Trustworthy Medical Artificial Intelligence,” BMC Medical Informatics and Decision Making 23, no. 1 (2023): 7. 36639799 10.1186/s12911-023-02103-9 PMC9840286

[49] D. Gödde , S. Nöhl , C. Wolf , et al., “A SWOT (Strengths, Weaknesses, Opportunities, and Threats) Analysis of ChatGPT in the Medical Literature: Concise Review,” Journal of Medical Internet Research 25 (2023): e49368. 37865883 10.2196/49368 PMC10690535

[50] T. Martín Noguerol , F. Paulano‐Godino , M. T. Martín‐Valdivia , C. O. Menias , and A. Luna , “Strengths, Weaknesses, Opportunities, and Threats Analysis of Artificial Intelligence and Machine Learning Applications in Radiology,” Journal of the American College of Radiology 16, no. 9 (2019): 1239–1247. 31492401 10.1016/j.jacr.2019.05.047

[51] P. Y. X. Miff , M. I. bin Al Nazir , N. T. J. Hussain , et al., “Readiness Towards Artificial Intelligence Among Undergraduate Medical Students in Malaysia,” Education in Medicine Journal 15 (2023): 49–60.

[52] D. S. Liu , J. Sawyer , A. Luna , et al., “Perceptions of US Medical Students on Artificial Intelligence in Medicine: Mixed Methods Survey Study,” JMIR Medical Education 8, no. 4 (2022): e38325. 36269641 10.2196/38325 PMC9636531

